# Prevalence of Asymptomatic SARS-CoV-2 Infection in Children and Adults in Marion County, Indiana

**DOI:** 10.7759/cureus.9794

**Published:** 2020-08-16

**Authors:** James Wood, Dibyadyuti Datta, Brenda L Hudson, Katrina Co, Sarah Tepner, Emily Hardwick, Chandy C John

**Affiliations:** 1 Pediatric Infectious Diseases, Indiana University School of Medicine, Indianapolis, USA; 2 Pediatrics, Indiana University School of Medicine, Indianapolis, USA; 3 Indiana Clinical and Translational Sciences Institute, Indiana University School of Medicine, Indianapolis, USA

**Keywords:** pediatric, child, transmission

## Abstract

Background and Objectives: Two community studies outside the US showed asymptomatic infection with severe acute respiratory syndrome coronavirus 2 (SARS-CoV-2) in adults, but not in children <10 years of age. In this study, we assessed the prevalence of asymptomatic SARS-CoV-2 infection in children and adults in Marion County, Indiana.

Methods: Individuals living in Marion County with no symptoms of coronavirus 2019 disease (COVID-19) within seven days of enrollment were eligible for this cross-sectional household study. Study kits were delivered to the participant’s residence for self-swabbing, picked up by the study team, and tested by polymerase chain reaction (PCR) for SAR-CoV-2 infection.

Results: Five hundred eleven nasal swabs were collected from 119 children and 392 adults ≥18 years of age. One participant (seven years of age) tested positive, for an overall study prevalence of 0.2% (95% CI 0, 0.6%). The participant had no known contact with a person with SARS-CoV-2 infection, and five family members tested negative for infection. The child and family members all tested negative for infection 10 and 20 days after the first test, and none developed symptoms of COVID-19 for 20 days after testing.

Conclusions: Asymptomatic SARS-CoV-2 infection can occur in children <10 years with no known COVID-19 exposure. Large cohort studies should be conducted to determine prevalence of asymptomatic infection and risk of transmission from asymptomatic infection in children and adults over time.

## Introduction

A growing body of evidence suggests that asymptomatic and pre-symptomatic cases may significantly contribute to the spread of severe acute respiratory syndrome coronavirus 2 (SARS-CoV-2), the virus responsible for coronavirus disease 2019 (COVID-19) [1-4]. It has been suggested that children are a reservoir for asymptomatic infection, but population-based studies in Iceland and Italy that assessed symptomatic and asymptomatic SARS-CoV-2 infection found no infections in children <10 years of age [3,5].

An understanding of the prevalence of asymptomatic SARS-CoV-2 infection in US communities is critical to designing effective infection prevention strategies. Data on asymptomatic infection in US children and adults in the community are not currently available. For this reason, we conducted a community-based study of the prevalence of asymptomatic SARS-CoV-2 infection in adults and children in Marion County, Indiana.

## Materials and methods

Study design, population, and setting 

We conducted a cross-sectional household study of prevalence of asymptomatic SARS-CoV2 infection, titled Tracing Asymptomatic COVID-19 Through Indianapolis Communities (TACTIC) in Marion County, Indiana. Marion County, which encompasses the city of Indianapolis, is the largest county in Indiana with a population of 954,670 in 2018, with 235,211 children <18 years of age. Individuals were eligible if they resided in a zip code within Marion County and did not show any of the following symptoms within seven days of enrollment: temperature >100.5^o^F (taken orally, axillary or rectally), new onset cough, new onset diarrhea, new onset shortness of breath, or new onset sore throat. Individuals were excluded if they had previously tested positive for SARS-CoV-2 by polymerase chain reaction (PCR), or were unable to provide informed consent. Participants were enrolled and tested between April 27, 2020 and May 15, 2020. This study was reviewed and approved by the Indiana University Institutional Review Board (IRB).

Subject screening and enrollment

**Figure 1:**
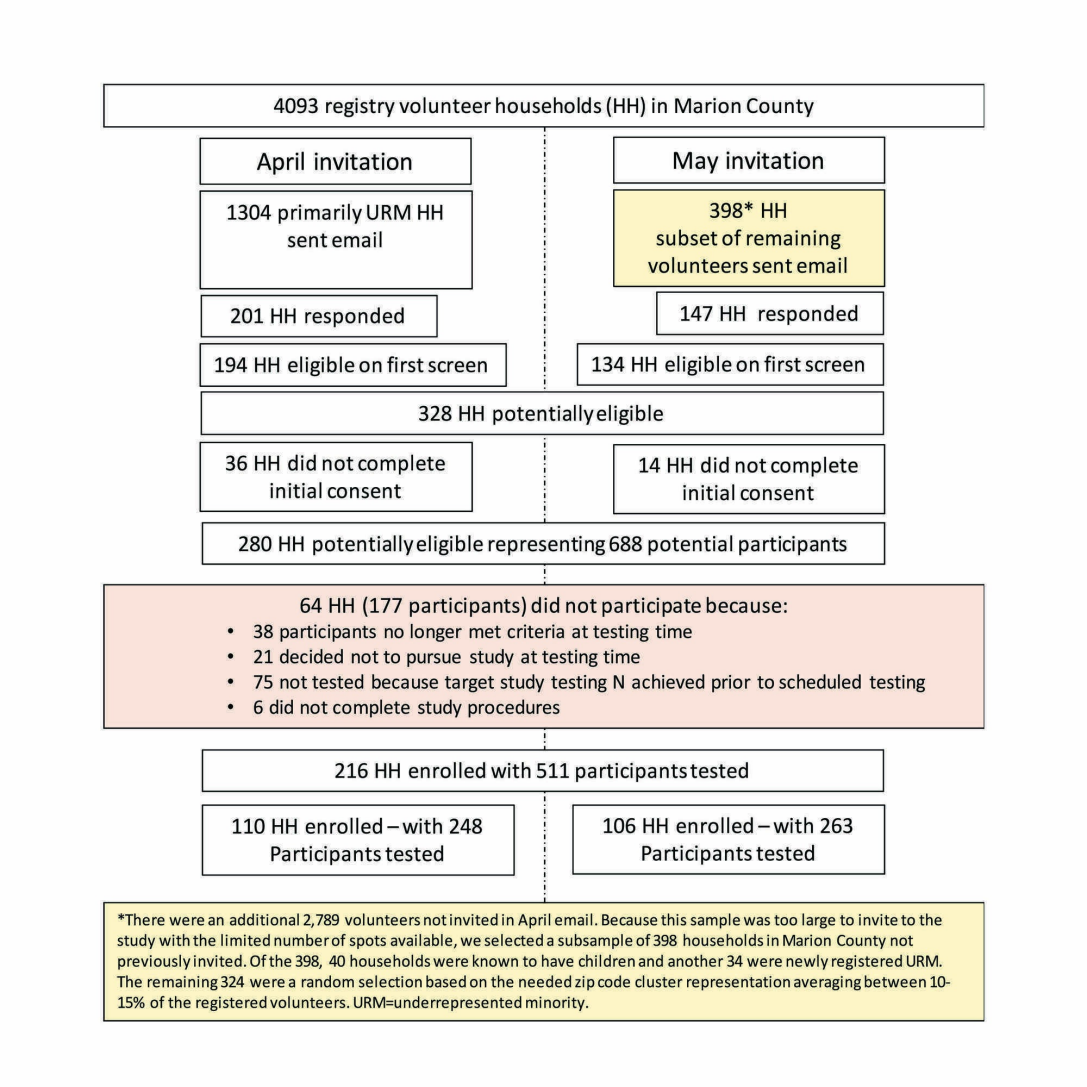
Flow chart of recruitment and enrollment strategy for the Tracing Asymptomatic COVID-19 Through Indianapolis Communities (TACTIC) study.

The study recruitment and enrollment strategy is outlined in Figure 1. Participants were recruited using a state-wide research registry, All IN for Health, which has 4,093 households in Marion County. A description of the study and directions on how to participate and enroll in the All IN for Health registry if interested were circulated through social media and local news outlets, including television and websites. Households with primarily underrepresented minorities (URM) were first invited to participate, and one week later, a subset of households was chosen according to the household having children, having newly registered URM, or according to the need for proportionate representation from 10 zip code clusters that comprised all of Marion County. Interested participants were sent an invitation to the screening survey using the secure, web-based software platform Research Electronic Data Capture (REDCap) hosted at Indiana University [6]. Eligible household members were then given a consent or assent (child aged 12-17) document to complete.

Sample collection and testing 

After obtaining informed consent, participants were directed to the study website with instructions and a video of nasal swab collection procedures [7]. Study participants were confirmed to be asymptomatic via REDCap prior to delivery of study kits with swabs and viral transport medium vials. After nasal swabbing by participants, sealed kits were picked up and swabs stored in a research laboratory at 4^o^C until transport within 24 hours to the Purdue Animal Disease Diagnostic Lab, a Clinical Laboratory Improvement Amendments (CLIA)-certified lab, for PCR testing. Viral RNA was isolated using the MagMax Viral/Pathogen Nucleic Acid Isolation Kit (Thermo Fisher Scientific, Waltham, MA) with a KingFisher™ Flex Magnetic Particle Processor with 96 Deep-Well Head (Thermo Fisher Scientific), and testing for SARS-CoV2 was done by real-time polymerase chain reaction (RT-PCR) using the TaqPath™ RT-PCR COVID-19 Kit (Thermo Fisher Scientific) with an Applied Biosystems™ 7500 Fast Real-Time PCR Instrument used with SDS Software v1.5.1 (Thermo Fisher Scientific) and the data analyzed and interpreted using the Applied Biosystems™ COVID-19 Interpretive Software (Thermo Fisher Scientific). This multiplex assay detects the ORF1ab, N gene, and S gene of the SARS-COV-2 virus. The kit also contains an exogenous extraction control (MS2 Phage Control) to confirm nucleic extraction and monitor for PCR inhibition. 

Statistical analysis

Based on previous studies finding a low overall community prevalence [3,5], the study sample size had the power to detect a 1% prevalence (95% confidence interval [CI] 0.1, 1.9%) with 95% confidence interval for proportion based on normal approximation of asymptomatic infection. All other study data were descriptive.

## Results

Characteristics of the study population

**Figure 2:**
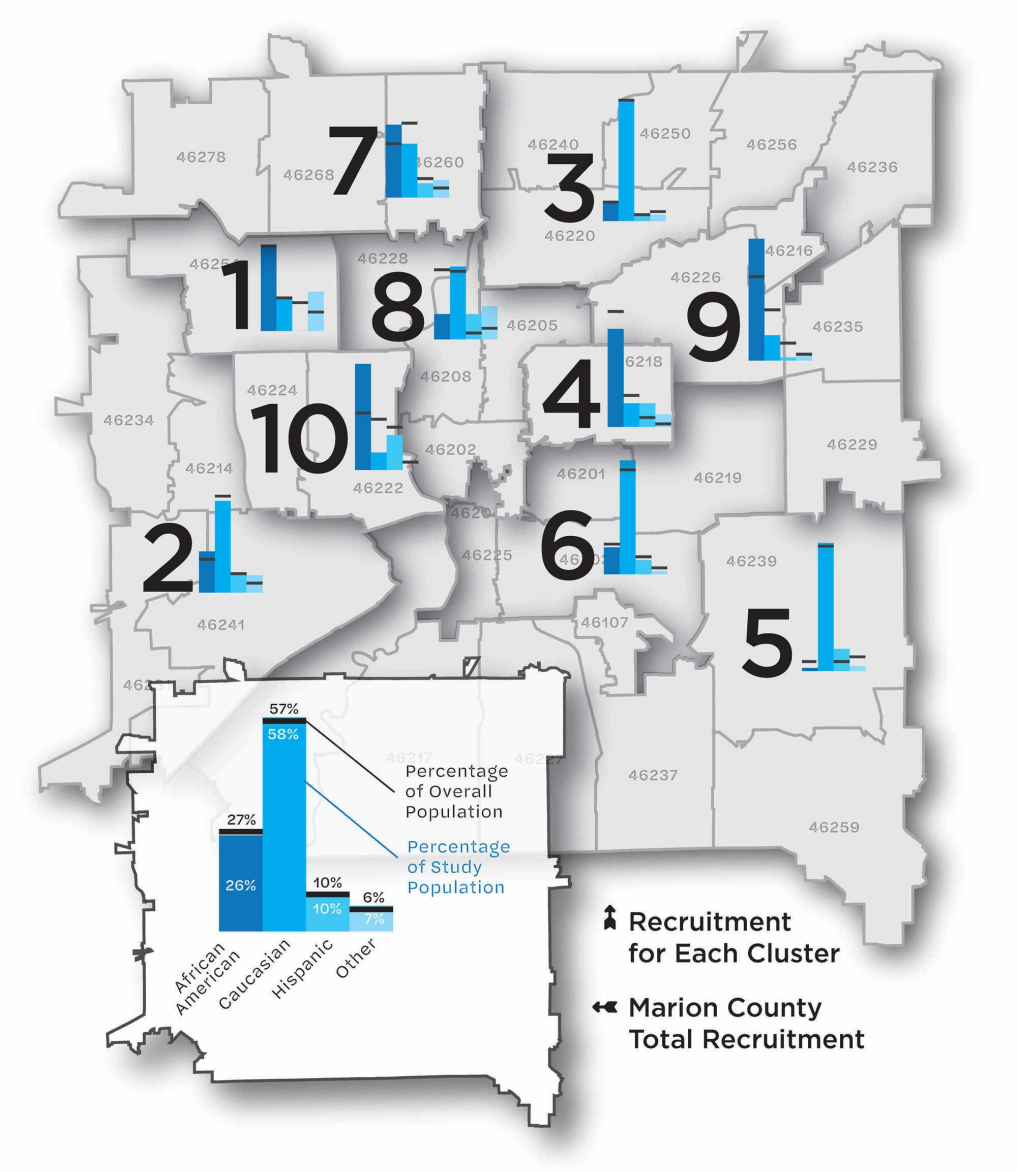
Map of Marion County, Indiana, by zip code cluster, and proportion of population within zip code cluster by race and ethnicity. Using American Community Survey data, zip codes with similar demographics were grouped into ten clusters. This figure shows a cluster’s racial and ethnic breakdown, in terms of overall population percentage (the black horizontal bar) and the study participant percentage (the blue vertical bars). Data and demographic analysis provided by The Polis Center at IUPUI.

Nasal swabs were collected on 511 participants, including 119 children <18 years of age, and 392 individuals ≥18 years of age. Distribution of county and study population by age group is shown in Table 1. Racial and ethnic characteristics of the study and county populations, by zip code cluster and overall, are shown in Figure 2. 

**Table 1:** Age distribution in study population compared to Marion County residents. ^a^ Based on stats.indiana.edu

N (%)	Study Population (N=511)	Marion County ^a^ (N=954,670)
Age, years (median)	36	34.5
Preschool (0 to 4)	22 (4.3)	68,903 (7.2)
School Age (5 to 17)	97 (19.0)	166,308 (17.4)
College Age (18 to 24)	30 (5.9)	88,123 (9.2)
Young Adult (25 to 44)	175 (34.2)	284,566 (29.8)
Older Adult (45 to 64)	136 (26.6)	226,412 (23.7)
Seniors (65 and older)	51 (10.0)	120,358 (12.6)

SARS-CoV-2 PCR testing results

Among the 511 participants tested, one participant, a seven-year-old boy, tested positive for SARS-CoV-2 (prevalence 0.2% [95% CI 0, 0.6%]). The prevalence among children (n=119) was 0.8% (95% CI 0, 2.5%). The one child who tested positive was biracial (African-American/Caucasian) and lived in a household with his mother and five siblings. One child declined study participation. The other four children and the mother were all negative for SARS-CoV2 infection. Neither the infected child nor anyone else in the household reported having contact with a person known to have COVID-19. The cycle threshold (Ct) values for the three target gene PCR reactions were 23-25, low levels indicating a significant viral load. Ten days after testing, the child, siblings, and mother all tested negative on repeat PCR testing 10 and 20 days after initial testing and all remained asymptomatic during this period. Among the 392 asymptomatic adults ≥18 years of age, none tested positive for SARS-CoV-2 infection (prevalence 0%).

## Discussion

Multiple studies from China have reported asymptomatic SARS-CoV-2 infection in children, but these studies involved children in contact with infected individuals and were not community surveys of asymptomatic infection [1,2]. Community surveys in Iceland and Italy that included symptomatic and asymptomatic individuals found a low prevalence of infection (0.8% to 2.6%), and both studies found no infection in children <10 years of age [3,4]. In the state of Indiana, a community prevalence study of individuals >12 years of age found an active infection rate (by PCR) of 1.7% and an overall prevalence of current (PCR) or previous infection (SARS-CoV-2 antibody) of 2.8% [8]. Prevalence of asymptomatic infection in this study was 0.76%, within the range of our finding of 0.2% prevalence in the population [8]. Despite the concern about spread of SARS-CoV-2 infection from asymptomatic individuals in the community, there have been no published studies of SARS-CoV-2 infection in asymptomatic adults and children 12 years and under in the United States to date.

In the present study, we found no asymptomatic infection in 392 adults ≥18 years of age, but found that one of 119 asymptomatic children ≤18 years was infected (prevalence 0.8%, 95% CI 0, 2.5%). The sole infected individual was seven years of age, demonstrating, contrary to prior studies, that children under 11 years of age in the community may carry asymptomatic infection. The source of the child’s infection was unclear, as no other household members tested positive. It is possible other family members in the home were previously infected and no longer shedding. Future serologic studies would be helpful and are planned. The positive result was reported to the Indiana State Department of Health for further evaluation and contact tracing.

The positive test rate in the state of Indiana during the study period was 11.3%, with an average of 591 positive individuals per day [9]. Our study's finding of a very low prevalence of asymptomatic infection provides reassurance on the benefits of the state implementation of social distancing measures, which went in to effect March 24th with a stay at home order and a phased reopening starting May 11th in Marion county. Even with this low prevalence, infection in one child out of 119 tested (0.84%) yields an estimate of 1977 children asymptomatically infected in Indianapolis/Marion County (95% CI 0, 6209). The large confidence intervals demonstrate the preliminary nature of this data. Larger studies of asymptomatic infection that include young children are needed to determine the relative prevalence of asymptomatic infection in children and adults with greater precision. Additionally, longitudinal studies across multiple geographic areas are needed to determine how prevalence varies in different areas, especially as social distancing measures are relaxed. Testing of antibodies to SARS-CoV-2 in these individuals could help to determine the rate of past infection, another important measure for which there is little data in children. Antibody testing is planned in this cohort. The increasing reports of multisystem inflammatory syndrome in children (MIS-C) temporally associated with prior SARS-CoV-2 infection, most of which appears to have been asymptomatic [10-12], underscore the importance and urgency of increasing our understanding of asymptomatic SARS-CoV-2 infection and its potential sequelae in children.

Strengths of this study include the specific investigation of asymptomatic individuals; use of community engagement to recruit a representative cohort of individuals; the innovative sample distribution and collection methods, which made the study possible in a period of active SARS-CoV-2 transmission; use of self/parent-administered nasal swabs, recently shown to be similar in sensitivity for SARS-CoV-2 detection to nasopharyngeal swabs [13,14], which made home testing of children feasible; and the intentional inclusion of both children and adults in testing. The primary limitations were the relatively small sample size for a population study and the non-random selection of study participants. Despite the small sample size, we were able to detect low frequency infection, but the 95% confidence intervals of the estimates included zero, so the study data are best viewed as important initial data on which to base future studies of asymptomatic infection in children and adults. We chose a non-random recruitment process because we had a research registry that mirrored overall population demographics and therefore was likely to provide a sample representative of the geographic and racial diversity of the county/city population. Additionally, study enrollment required access to internet/email which may have biased our population to a higher socioeconomic class. Random selection might have derived a population more fully representative of the county population, but participation rates would likely have been far lower, and the final sample may not have been substantially different from the sample we obtained.

## Conclusions

In the present study, we found asymptomatic SARS-CoV-2 infection in one seven-year-old child with no history of known SARS-CoV-2 exposure, out of 119 children tested (prevalence 0.84%, 95% CI, 0, 2.5%) and in none of the 392 adults ≥18 years of age tested. The study demonstrates that children <10 years of age with no known SARS-CoV-2 exposure may harbor asymptomatic SARS-CoV-2 infection. In this instance the child did not transmit infection to close contacts. The study sets the stage for future larger studies of asymptomatic infection in children to determine risk and patterns of transmission in the community and devise strategies for infection prevention.
